# Iron chelators in breast cancer therapy: mechanisms and clinical applications – a narrative review

**DOI:** 10.1097/MS9.0000000000003296

**Published:** 2025-04-25

**Authors:** Emmanuel Ifeanyi Obeagu, Anthonia Onyinye Ngwoke, Garikai Malunga

**Affiliations:** a Department of Biomedical and Laboratory Science, Africa University, Zimbabwe; b Department of Human Physiology, Faculty of Basic Medical Sciences, Enugu State University of Science and Technology, Enugu, Nigeria

**Keywords:** breast cancer therapy, clinical applications, iron chelators, mechanisms, targeted therapy

## Abstract

Iron is an essential element for cell growth and metabolism, but its dysregulation is a key feature in the pathogenesis of various cancers, including breast cancer. Cancer cells require elevated iron levels to support their rapid growth, and as such, iron chelation has emerged as a promising therapeutic strategy. Iron chelators work by binding free iron in cancer cells, preventing its use in critical biological processes and thereby disrupting tumor cell proliferation. This review discusses the mechanisms of action of iron chelators in breast cancer therapy, highlighting how they induce oxidative stress, impair DNA repair, and alter cellular iron homeostasis, ultimately leading to cancer cell death. Iron chelation therapy has been explored in several clinical and preclinical studies for its potential to enhance the effectiveness of conventional breast cancer treatments, including chemotherapy and radiotherapy. By depleting intracellular iron, iron chelators can sensitize cancer cells to these treatments, increasing the cytotoxic effects and improving patient outcomes. Additionally, novel iron chelators with higher specificity for tumor cells are being developed, which aim to minimize off-target effects and enhance therapeutic efficacy. While iron chelation therapy has shown promise in early-phase trials, further research is needed to optimize these agents for clinical use in breast cancer treatment.

## Introduction

Iron is a crucial element for various cellular processes, including DNA synthesis, mitochondrial function, and cellular respiration. It plays a central role in maintaining the cell’s metabolic activities and supporting growth and division. In normal cells, iron is tightly regulated through complex mechanisms that ensure an adequate supply while preventing its excess, which can result in oxidative stress and cellular damage. However, cancer cells, including those in breast cancer, often exhibit altered iron metabolism, with increased iron uptake and reduced storage or efflux, facilitating their rapid growth and survival in the tumor microenvironment. Dysregulated iron homeostasis in tumors has become a hallmark of cancer biology, and its potential for therapeutic targeting is an area of significant interest^[1,2]^. Breast cancer is one of the most prevalent cancers worldwide, accounting for a substantial number of cancer diagnoses and deaths. The disease is heterogeneous, with various subtypes that differ in clinical behavior, prognosis, and response to treatment. While many advances have been made in breast cancer treatment, including surgery, chemotherapy, radiation therapy, and targeted therapies, the prognosis for patients with advanced or metastatic breast cancer remains poor. Resistance to conventional therapies is a major challenge, highlighting the need for new therapeutic strategies. Iron chelation has emerged as a promising approach, as it targets a key metabolic process in cancer cells – iron metabolism – that is often dysregulated in tumors^[3,4]^. Iron chelation refers to the process of binding free, unbound iron in the body using specific chelating agents^[5]^. The role of iron in cancer is multifaceted. Iron acts as a cofactor for various enzymes that participate in cellular respiration, DNA synthesis, and redox reactions. In cancer cells, which have an elevated rate of metabolism due to rapid cell division, iron is crucial for sustaining these high metabolic demands. Furthermore, the ability of iron to catalyze the production of reactive oxygen species (ROS) through the Fenton reaction can lead to oxidative stress, a key driver of cancer progression. Tumor cells often manipulate iron metabolism to increase iron uptake while limiting iron export, creating a microenvironment that supports tumor growth. This dysregulation of iron homeostasis in cancer cells has prompted researchers to explore iron chelation as a potential therapeutic intervention to reverse this process and inhibit cancer cell proliferation^[6,7]^. Iron chelators are small molecules that bind iron and prevent its participation in essential biological reactions. The most well-known and widely studied iron chelators are deferoxamine (DFO), deferasirox, and deferiprone. These agents have been extensively used in the treatment of iron overload disorders, such as thalassemia, but their application in cancer therapy is a newer and rapidly expanding area of research^[8,9]^.

**HIGHLIGHTS**

- Tumor suppression: Iron chelators deplete intracellular iron, hindering breast cancer cell growth and survival.
- Reactive oxygen species modulation: Chelators reduce reactive oxygen species, limiting DNA damage and tumor progression.
- Ferroptosis activation: Iron chelators induce ferroptosis, a targeted cancer cell death pathway.
- Synergistic effects: Combined with chemotherapy or radiotherapy, iron chelators enhance treatment efficacy.
- Clinical potential: Agents like deferoxamine and deferasirox show promise in preclinical and early clinical trials.

Iron chelation therapy’s ability to sensitize tumors to chemotherapy and radiation therapy makes it a promising addition to the arsenal of breast cancer treatment options. Many chemotherapeutic agents, such as doxorubicin and paclitaxel, rely on oxidative stress and DNA damage to kill cancer cells. Iron chelation can enhance the cytotoxic effects of these drugs by increasing oxidative stress and reducing the repair of DNA damage, which may overcome the resistance seen in some breast cancer subtypes. In combination with radiation therapy, iron chelation can increase the generation of ROS, enhancing the damage caused by radiation and improving the efficacy of this treatment. These synergistic effects of iron chelation with conventional therapies highlight its potential to improve clinical outcomes in breast cancer^[10,11]^. In addition to improving the efficacy of conventional treatments, iron chelation can also influence other aspects of cancer biology, such as immune response, cell metabolism, and tumor microenvironment dynamics. Cancer cells rely on altered metabolic pathways, including increased glycolysis and mitochondrial dysfunction, to support their rapid growth. Iron is a key regulator of these metabolic pathways, and its chelation can disrupt the metabolic balance of tumor cells, further impeding their survival. Moreover, iron chelation can affect the tumor microenvironment by influencing immune cell activity, angiogenesis, and extracellular matrix remodeling, which are all crucial processes for tumor progression and metastasis^[12,13]^. The potential clinical application of iron chelation therapy in breast cancer is a rapidly evolving area of research. Preclinical studies and early-phase clinical trials have provided encouraging evidence for the role of iron chelators in enhancing the effectiveness of breast cancer treatments^[1,14]^.

Iron metabolism plays a critical role in the progression and survival of breast cancer cells. Given that cancer cells, including breast cancer cells, often exhibit dysregulated iron homeostasis to support rapid proliferation, targeting iron availability presents an innovative approach to disrupting the cancerous growth cycle. Iron chelation therapy, which aims to reduce intracellular iron levels, has garnered significant attention as a potential therapeutic strategy in cancer management. However, while iron chelation shows promise in preclinical studies, its clinical application in breast cancer therapy remains underexplored and needs further investigation^[14,15]^. This review is justified by the need to consolidate the current research on the mechanisms of action of iron chelators, their potential for enhancing the effects of conventional treatments such as chemotherapy and radiotherapy, and their clinical outcomes in breast cancer patients. By synthesizing available data, this review aims to provide a comprehensive understanding of how iron chelators could contribute to improved breast cancer therapy, especially in cases of treatment resistance or metastatic disease. Furthermore, as personalized medicine becomes increasingly important in cancer care, understanding the role of iron metabolism in different breast cancer subtypes and tailoring iron chelation therapy accordingly could significantly impact clinical practice^[16,17]^. Additionally, while there are existing studies exploring the potential of iron chelation in cancer therapy, there remains a gap in knowledge regarding the optimal use of iron chelators, their side effects, and their integration with other therapeutic modalities^[18]^.

The aim of this review is to explore the role of iron chelation therapy in breast cancer treatment by investigating the mechanisms underlying iron metabolism dysregulation in cancer cells, the therapeutic potential of various iron chelators, and their clinical applications in breast cancer therapy.

## Review methods

### Literature search strategy

A comprehensive literature search was conducted using databases such as PubMed, Google Scholar, Scopus, and Web of Science. The search was restricted to articles published to capture the most up-to-date research on iron chelation therapy in breast cancer. The search terms included combinations of keywords such as “iron chelation,” “breast cancer,” “therapy,” “deferoxamine,” “deferasirox,” “iron metabolism,” and “chemotherapy.” Studies published in English and peer-reviewed journals were considered eligible for inclusion.

### Inclusion and exclusion criteria

Articles were selected based on predefined inclusion and exclusion criteria. Inclusion criteria encompassed:

- Original research articles, clinical trials, systematic reviews, and preclinical studies focused on the role of iron chelators in breast cancer therapy.
- Studies investigating the mechanisms of iron metabolism in cancer cells and their impact on tumor progression.
- Articles exploring the clinical application, efficacy, and safety of iron chelation therapies, either as monotherapies or in combination with other treatments.

Exclusion criteria were:

- Studies that did not address the use of iron chelators in breast cancer.
- Non-peer-reviewed articles, abstracts, or conference proceedings.
- Studies on non-cancerous conditions or iron chelation in diseases other than cancer.

### Mechanisms of action of iron chelators in breast cancer

Iron chelators exert their effects by binding free iron in the body or within cells, preventing its participation in essential biological processes. The disruption of iron homeostasis induced by these agents leads to a variety of cellular consequences, particularly in rapidly proliferating tumor cells like those found in breast cancer. In breast cancer, iron is crucial for several key biological functions, including DNA replication, mitochondrial function, and cellular respiration. Iron chelators target these essential functions to inhibit tumor cell growth and survival, making them a potential therapeutic strategy for this malignancy^[19]^. One of the most significant mechanisms by which iron chelators act is through the induction of oxidative stress. Iron plays a critical role in cellular redox reactions, particularly in the Fenton reaction, where iron catalyzes the production of ROS. ROS are highly reactive molecules that can damage cellular components such as lipids, proteins, and DNA. In cancer cells, which are already under heightened oxidative stress due to their rapid growth and metabolic activity, iron chelation exacerbates this stress by limiting the availability of iron to participate in these reactions. The accumulation of ROS causes damage to cellular structures and DNA, leading to cell cycle arrest, apoptosis, or necrosis, thus impairing cancer cell survival^[20]^. In addition to increasing oxidative stress, iron chelators disrupt DNA synthesis and repair. Iron is a cofactor for ribonucleotide reductase, an enzyme essential for DNA synthesis. By binding to iron and reducing its availability, iron chelators inhibit ribonucleotide reductase activity, which leads to the depletion of deoxyribonucleotides required for DNA replication. This results in DNA damage and replication stress, a state that is especially detrimental to rapidly proliferating cancer cells. Moreover, iron is involved in DNA repair mechanisms, including base excision repair and double-strand break repair. The reduction of available iron impairs these repair processes, further promoting the accumulation of genetic mutations and increasing the sensitivity of cancer cells to chemotherapy and radiation therapy^[21]^.

Iron chelation can also affect the mitochondrial function of breast cancer cells. Mitochondria are the powerhouses of the cell, and they rely heavily on iron for the proper functioning of enzymes involved in the electron transport chain and ATP production. By chelating iron, iron chelators disrupt mitochondrial respiration, leading to mitochondrial dysfunction. This dysfunction not only reduces cellular energy production but also increases mitochondrial ROS generation, further exacerbating oxidative stress. Mitochondrial dysfunction can trigger a cascade of events, including activation of apoptotic pathways and mitochondrial membrane permeabilization, contributing to the death of cancer cells. Thus, iron chelation not only impairs tumor cell metabolism but also serves as an effective strategy to induce cell death^[22]^. Another important mechanism of action of iron chelators is their impact on cellular signaling pathways involved in tumor progression. Iron is known to regulate several key signaling pathways, including those involved in cell proliferation, survival, and angiogenesis. One of the most notable pathways influenced by iron is the hypoxia-inducible factor 1-alpha (HIF-1α) pathway, which plays a critical role in the cellular response to low oxygen levels (hypoxia). HIF-1α regulates genes involved in angiogenesis, metabolism, and cell survival. Under normal oxygen conditions, iron-dependent prolyl hydroxylase enzymes degrade HIF-1α. However, when iron is chelated, this degradation is impaired, leading to increased HIF-1α stability. The altered HIF-1α signaling can result in a metabolic shift in cancer cells, making them more susceptible to the effects of chemotherapy and radiation. Iron chelation also influences other signaling molecules such as NF-κB, which is involved in inflammation and cancer progression, contributing to the broad anti-tumor effects of iron chelators^[23]^.

Iron chelation therapy also affects the tumor microenvironment, which plays a critical role in cancer progression, metastasis, and resistance to therapy. Iron accumulation in the tumor microenvironment is known to promote angiogenesis, the formation of new blood vessels that supply nutrients and oxygen to growing tumors. Iron chelators inhibit angiogenesis by reducing the availability of iron for endothelial cells, which are essential for the formation of new blood vessels. Additionally, iron chelation can disrupt the functions of immune cells within the tumor microenvironment. For instance, macrophages, which often play a role in tumor progression, require iron for their activation and function. By depleting iron, chelators impair the immune response, potentially enhancing the anti-tumor immune response and sensitizing tumors to immune-based therapies^[24]^. Another key mechanism of action of iron chelators is their ability to sensitize breast cancer cells to chemotherapy and radiotherapy. Many chemotherapeutic agents, such as doxorubicin, paclitaxel, and cisplatin, rely on inducing oxidative stress and DNA damage to kill cancer cells. Iron chelators enhance the efficacy of these agents by further increasing oxidative stress, inhibiting DNA repair mechanisms, and reducing the tumor cells’ ability to repair chemotherapy-induced damage. In the context of radiation therapy, iron chelation enhances the production of ROS, which contributes to the DNA damage caused by radiation. By boosting the effects of chemotherapy and radiotherapy, iron chelation may help overcome resistance mechanisms that typically reduce the effectiveness of these treatments^[25]^.

**Figure 1.**
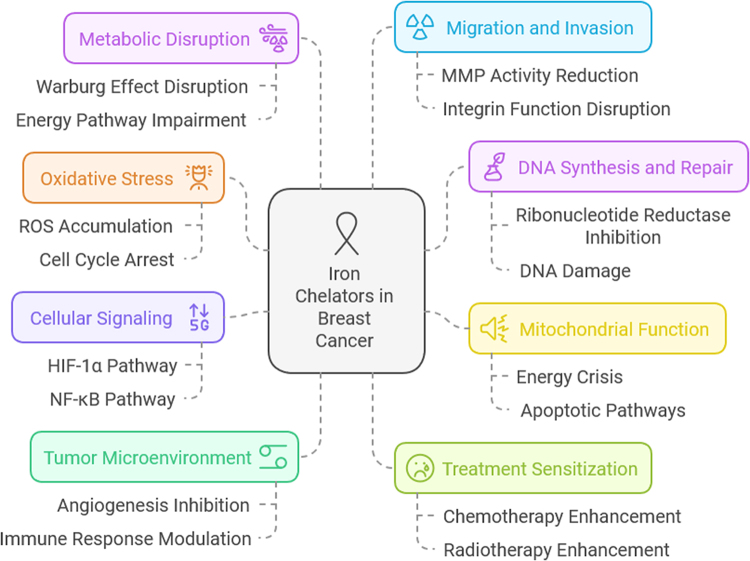
Mechanisms of action of iron chelators in breast cancer.

Additionally, iron chelators can alter tumor cell metabolism in a way that makes cancer cells more vulnerable to treatment. Cancer cells typically exhibit altered metabolic pathways, such as the Warburg effect, where they rely more on glycolysis rather than oxidative phosphorylation for energy production. This altered metabolism is iron-dependent, as iron is required for the activity of enzymes involved in both glycolysis and oxidative phosphorylation. By chelating iron, these metabolic pathways are disrupted, leading to an energy crisis in tumor cells that impairs their ability to proliferate and survive. The metabolic disruption caused by iron chelation can also render tumor cells more sensitive to other therapeutic interventions^[26]^. Finally, iron chelators impact tumor cell migration and invasion, which are crucial steps in metastasis. Iron is known to modulate the expression of matrix metalloproteinases (MMPs), enzymes that degrade the extracellular matrix and facilitate tumor cell invasion. Iron chelation can reduce MMP activity, thereby preventing the ability of breast cancer cells to invade surrounding tissues and metastasize to distant organs. Additionally, iron chelation disrupts the function of integrins and other cell adhesion molecules that play a role in cell migration, further inhibiting tumor metastasis^[4]^. Figure 1 shows mechanisms of action of iron chelators in breast cancer (provided by the authors).

### Effect of iron chelators in different breast cancer subtypes

Breast cancer is a heterogeneous disease with multiple histological subtypes, each exhibiting distinct molecular and clinical characteristics. Among these, invasive carcinoma of no special type (NST), invasive lobular carcinoma, mucinous carcinoma, and medullary carcinoma represent well-known subtypes. Iron chelators have shown promise in targeting breast cancer by disrupting iron metabolism, but their effects vary across different histological subtypes due to differences in tumor biology, iron dependency, and response to oxidative stress^[7,27]^.

### Invasive carcinoma of no special type (NST)

Invasive carcinoma of NST, formerly known as invasive ductal carcinoma, accounts for the majority of breast cancer cases. NST is characterized by significant heterogeneity, with subpopulations demonstrating varying degrees of iron dependency. Studies have revealed that NST tumors often exhibit overexpression of transferrin receptor 1 (TfR1) and ferritin, indicating high iron uptake and storage. This reliance on iron metabolism makes NST highly susceptible to iron chelation therapy. Iron chelators such as DFO and Dp44mT have been shown to induce apoptosis in NST by inhibiting iron-dependent DNA synthesis and promoting mitochondrial dysfunction. Additionally, iron chelation reduces ROS levels, which are critical for NST tumor proliferation. Given the aggressive nature of some NST cases, combination therapy with iron chelators and conventional chemotherapy has demonstrated enhanced anti-tumor effects, particularly in triple-negative NST, where iron metabolism is even more dysregulated^[28,29]^.

### Invasive lobular carcinoma (ILC)

Invasive lobular carcinoma (ILC) is the second most common histological subtype of breast cancer, often characterized by loss of E-cadherin expression, leading to diffuse infiltration of tumor cells. Unlike NST, ILC tends to have lower proliferative rates and distinct molecular pathways governing tumor progression. While ILC is not traditionally associated with high iron dependency, recent studies suggest that iron metabolism plays a role in its progression, particularly through oxidative stress-mediated DNA damage. Iron chelators, particularly deferasirox (DFX), have demonstrated the ability to modulate oxidative stress in ILC models, leading to reduced tumor cell viability. Furthermore, since ILC is often hormone receptor-positive, iron chelation combined with endocrine therapy may provide an avenue for enhanced treatment response. However, more research is needed to establish the efficacy of iron chelators in ILC, given its unique tumor biology^[30,31]^.

### Mucinous carcinoma

Mucinous carcinoma of the breast is a rare subtype characterized by extracellular mucin production, which creates a distinct tumor microenvironment (TME). This mucin-rich environment has been proposed to act as a barrier to drug penetration, potentially affecting the efficacy of iron chelators. Interestingly, mucinous carcinoma exhibits lower levels of proliferation compared to NST, but iron-dependent pathways still contribute to its progression. DFO and deferiprone have shown moderate efficacy in mucinous carcinoma models by reducing intracellular iron stores and impairing cell cycle progression. However, the dense mucinous stroma may limit drug diffusion, suggesting that nanoparticle-based delivery systems for iron chelators could improve therapeutic outcomes^[29]^.

### Medullary carcinoma

Medullary carcinoma of the breast is an uncommon but aggressive subtype often associated with an immune-rich microenvironment and increased expression of immune checkpoint proteins. Unlike other breast cancer subtypes, medullary carcinoma exhibits extensive lymphocytic infiltration, which may influence its response to iron chelation therapy. Studies indicate that medullary carcinoma cells exhibit high ferritin expression, suggesting an active role of iron metabolism in their growth. Dp44mT, a highly potent iron chelator, has demonstrated cytotoxic effects in medullary carcinoma by promoting ferroptosis – an iron-dependent form of cell death. Additionally, iron chelators may indirectly enhance anti-tumor immunity in medullary carcinoma by modulating the immune landscape and reducing tumor-promoting macrophages^[32]^.

### Functions of iron chelators in breast cancer: tumor cells and the microenvironment

Iron chelators have emerged as a promising therapeutic approach in breast cancer due to their ability to disrupt iron homeostasis, a critical factor in tumor progression. While cancer cells heavily rely on iron for proliferation and survival, the TME also plays a crucial role in supporting cancer growth, angiogenesis, and immune evasion. By targeting both tumor cells and their surrounding microenvironment, iron chelators exhibit multifaceted anti-cancer effects^[33]^.

1. Effects of iron chelators on tumor cells: Breast cancer cells exhibit a heightened demand for iron due to its essential role in DNA synthesis, mitochondrial function, and redox balance^[27]^. Iron chelators exert their effects on tumor cells through several key mechanisms:

- Inhibition of proliferation and cell cycle arrest: Iron chelators such as DFO and deferasirox reduce intracellular iron availability, leading to the inhibition of ribonucleotide reductase, an enzyme required for DNA synthesis. This results in cell cycle arrest, primarily at the G1/S phase, restricting tumor growth.
- Induction of apoptosis and ferroptosis: By depriving cancer cells of iron, iron chelators trigger mitochondrial dysfunction, leading to the activation of caspase-mediated apoptosis. Additionally, some chelators, such as Dp44mT, induce ferroptosis, an iron-dependent form of programmed cell death characterized by lipid peroxidation and oxidative stress.
- Inhibition of metastasis and invasion: Iron is crucial for the activity of MMPs, enzymes that degrade the extracellular matrix and facilitate tumor invasion. Iron chelators inhibit MMP activity, thereby reducing the ability of breast cancer cells to invade surrounding tissues and metastasize.
- Suppression of iron-driven oncogenic pathways: Cancer cells exploit iron-related pathways to sustain their survival, including the overexpression of TfR1 for enhanced iron uptake. Iron chelators disrupt these pathways, leading to downregulation of oncogenic signaling, such as c-Myc and HIF-1α, both of which promote tumor aggressiveness.

2. Effects of iron chelators on the TME: Beyond targeting tumor cells directly, iron chelators also modulate the tumor microenvironment, which consists of immune cells, fibroblasts, blood vessels, and extracellular matrix components that support cancer progression^[34]^.

- Reduction of tumor angiogenesis: The tumor microenvironment relies on angiogenesis to supply oxygen and nutrients to rapidly growing cancer cells. Iron chelators, particularly DFO, inhibit angiogenesis by downregulating vascular endothelial growth factor (VEGF) and reducing HIF-1α expression, thereby impairing new blood vessel formation.
- Modulation of immune responses: The immune landscape within the TME is highly influenced by iron metabolism. Tumor-associated macrophages (TAMs), which often promote tumor growth, thrive in iron-rich environments. Iron chelation alters macrophage polarization, shifting them from a pro-tumor M2 phenotype to an anti-tumor M1 phenotype, thereby enhancing immune-mediated tumor suppression.
- Disruption of cancer-associated fibroblasts (CAFs): CAFs contribute to tumor progression by secreting growth factors and extracellular matrix components that create a protective niche for cancer cells. Iron chelators reduce CAF activity by interfering with iron-dependent metabolic pathways, thereby weakening the structural and biochemical support of the tumor.
- Alteration of the oxidative stress balance: While iron-induced ROS can promote tumorigenesis, excessive ROS levels can also be cytotoxic to cancer cells. Iron chelators modulate ROS production within the TME, limiting oxidative damage that supports cancer cell survival while simultaneously inducing stress that leads to tumor cell death.

- Recent research trends on accumulating evidence for environmental and life style factors that influences disease mechanisms and progression in human populations: Recent research has increasingly highlighted the profound impact of environmental and lifestyle factors on disease mechanisms and progression in human populations. These factors, encompassing diet, physical activity, exposure to pollutants, and socioeconomic conditions, interact intricately with genetic predispositions, influencing the onset and trajectory of various diseases^[35]^.

- Diet and nutrition: Dietary habits have undergone significant changes over recent decades, with a notable shift toward high consumption of processed foods rich in fats and sugars. This nutritional transition has been linked to a rise in non-communicable diseases, including obesity, type 2 diabetes, and certain cancers. For instance, the increasing incidence of colorectal cancer among young adults has been partially attributed to dietary factors, with studies suggesting that those born after the 1960s face higher risks due to environmental changes rather than genetic factors^[36]^.

- Physical activity and sedentary lifestyle: The modern lifestyle, characterized by prolonged periods of inactivity, contrasts sharply with the physically active lives of our ancestors. This sedentary behavior has been associated with an increased risk of chronic diseases such as cardiovascular ailments and metabolic disorders. Regular physical activity is known to induce beneficial epigenetic modifications, enhancing gene expression profiles that protect against inflammation and oxidative stress. Conversely, lack of exercise can lead to adverse epigenetic changes, promoting disease development^[37]^.

- Exposure to environmental pollutants: Environmental pollutants, including air pollution and chemical contaminants, have been implicated in the pathogenesis of various diseases. Exposure to pollutants can lead to epigenetic modifications, such as DNA methylation changes, which alter gene expression and increase disease susceptibility. For example, air pollution has been linked to decreased DNA methylation levels, potentially contributing to the development of conditions like lung cancer and asthma^[38]^.

- Socioeconomic factors and stress: Socioeconomic disparities significantly influence health outcomes, with stress and trauma being well-established risk factors for diseases ranging from heart disease to mental health disorders. Chronic stress, often prevalent in lower socioeconomic settings, can lead to epigenetic alterations that affect gene expression related to stress responses and immune function, thereby increasing disease risk^[39]^.

- Gene–environment interactions: The interplay between genetic predispositions and environmental exposures, known as gene–environment interactions, plays a crucial role in disease development. Environmental factors can influence gene expression through epigenetic mechanisms, thereby modifying an individual’s susceptibility to diseases. For instance, exposure to certain environmental triggers can lead to epigenetic changes that either suppress or activate genes associated with disease risk^[38]^.

- Emerging concerns: Alarmingly, there has been a notable increase in early-onset diseases, particularly cancers, among younger populations. Research indicates that individuals born in the 1980s and 1990s have higher risks for several cancers, including breast, pancreas, kidney, and liver cancers. This trend suggests that lifestyle and environmental factors, rather than genetic changes, are contributing to the rising incidence of these diseases in younger cohorts^[39]^.

### Integrating lifestyle, environmental factors, and personalized disease biomarkers: a holistic approach to precision medicine

The study of lifestyle, environmental exposures, and genetic predisposition has become increasingly relevant in understanding disease mechanisms and progression. While factors such as diet, physical activity, stress, and pollution significantly impact health outcomes, personalized biomarker analysis provides a deeper, molecular-level understanding of disease risk and progression. Integrating these domains is essential for developing precision medicine approaches that tailor prevention and treatment strategies to an individual’s unique profile^[40]^.

### Lifestyle and environmental influences on health

Human health is shaped by complex interactions between genetics, lifestyle choices, and environmental exposures. These factors influence disease risk through mechanisms such as epigenetic modifications, oxidative stress, chronic inflammation, and immune dysregulation^[41]^.

1. Diet and nutrition: Poor dietary choices can lead to metabolic imbalances, obesity, and gut microbiome alterations, increasing susceptibility to diseases such as diabetes, cardiovascular disorders, and cancer. Nutritional biomarkers (e.g., omega-3 fatty acids, vitamin D levels) can provide insights into dietary deficiencies or excesses affecting disease risk^[42]^.
2. Physical activity and sedentary behavior: Regular exercise induces protective epigenetic changes, improving mitochondrial function and reducing systemic inflammation. Biomarkers such as circulating cytokines, lactate thresholds, and insulin sensitivity markers can assess individual responses to exercise interventions^[43]^.
3. Environmental pollutants and toxins: Exposure to air pollution, pesticides, heavy metals, and endocrine disruptors can lead to DNA methylation changes, immune dysfunction, and increased oxidative stress, predisposing individuals to chronic diseases. Biomarkers such as heavy metal levels, oxidative stress markers, and inflammatory cytokines can reveal the impact of environmental exposures on health^[44]^.
4. Psychosocial stress and mental health: Chronic stress alters the hypothalamic-pituitary-adrenal (HPA) axis, affecting immune responses and metabolic health. Biomarkers such as cortisol levels, inflammatory markers (IL-6, CRP), and brain-derived neurotrophic factor (BDNF) can assess the physiological impact of stress^[45]^.

### The role of personalized disease biomarkers

Biomarkers serve as measurable indicators of biological processes, disease states, or responses to treatment. By integrating biomarker data with lifestyle and environmental studies, a personalized health approach can be developed.

Key types of personalized biomarkers include:

- Genomic biomarkers: Identify genetic predispositions to diseases (e.g., BRCA1/2 mutations in breast cancer).
- Epigenetic biomarkers: Reflect lifestyle and environmental influences on gene expression (e.g., DNA methylation changes linked to air pollution or smoking).
- Metabolic biomarkers: Assess metabolic function and disease risk (e.g., glucose tolerance, lipid profiles, insulin resistance markers).
- Inflammatory biomarkers: Indicate chronic disease risk due to lifestyle factors (e.g., C-reactive protein (CRP), IL-6, TNF-α).
- Microbiome biomarkers: Reveal gut microbial imbalances related to diet, medication, and disease susceptibility.

For example, an individual with a high genetic risk for diabetes (e.g., TCF7L2 gene variant) may have their blood sugar regulation biomarkers monitored while also assessing their dietary habits and physical activity levels. This integrated approach allows for early intervention strategies tailored to that individual’s specific risk factors^[46]^.

### Bridging the gap: toward precision medicine

To optimize disease prevention and treatment, a multidimensional approach that integrates lifestyle, environment, and personalized biomarkers is needed. This approach enables:

1. Early disease detection: Identifying individuals at risk based on biomarker patterns influenced by lifestyle and environmental factors. Detecting pre-cancerous changes through methylation biomarkers linked to smoking and air pollution exposure.
2. Personalized intervention strategies: Tailoring treatments based on an individual’s unique biochemical and genetic profile. Adjusting diet and supplementation based on nutritional biomarker levels rather than generalized dietary guidelines.
3. Predictive health modeling: Using machine learning and AI to analyze longitudinal biomarker data, lifestyle trends, and environmental exposures to predict disease outcomes. AI-driven risk prediction models for cardiovascular diseases integrating lifestyle factors (diet, exercise) with genetic and metabolic markers.
4. Targeted preventive strategies: Developing precision public health initiatives based on population-wide biomarker analyses of environmental and lifestyle factors. Creating pollution-specific intervention programs based on oxidative stress biomarker trends in urban populations.

### Molecular pathological epidemiology in breast cancer: investigating lifestyle factors and biomarkers for risk and clinical outcomes

Breast cancer, one of the most prevalent malignancies worldwide, arises from a complex interplay between genetic predisposition, environmental exposures, and lifestyle factors. Traditional epidemiology has long established risk factors such as obesity, physical inactivity, alcohol consumption, smoking, and dietary patterns, while advances in molecular oncology have identified tumor-specific genetic and epigenetic alterations. However, the integration of these domains remains a growing challenge. Molecular pathological epidemiology (MPE) is an emerging field that bridges epidemiological data with molecular pathology, enabling a more nuanced understanding of how lifestyle and environmental factors influence breast cancer development and progression at the molecular level. MPE provides a framework for investigating biomarkers associated with lifestyle risk factors, ultimately allowing for improved risk assessment, early detection, and personalized treatment strategies.^[47-49]^

### The role of molecular pathological epidemiology in breast cancer research

MPE research seeks to correlate lifestyle exposures with specific molecular subtypes of breast cancer, revealing how external factors influence disease etiology and progression. This approach involves:

1. Identifying biomarkers linked to lifestyle and environmental factors:

- Epigenetic modifications: DNA methylation changes due to smoking, air pollution, or dietary habits can regulate oncogene and tumor suppressor expression.
- Inflammatory biomarkers: Chronic inflammation, influenced by obesity and diet, is associated with increased cytokine levels (e.g., IL-6, TNF-α, CRP) that promote tumorigenesis^[50]^.
- Metabolic biomarkers: Insulin resistance, hyperglycemia, and dyslipidemia, often linked to poor diet and sedentary behavior, influence breast cancer progression through the PI3K/AKT pathway.

2. Examining the impact of lifestyle factors on breast cancer subtypes:

- Breast cancer is classified into different molecular subtypes based on receptor expression:
  - Hormone receptor-positive (HR) + breast cancer (Luminal A/B)
  - HER2-enriched breast cancer
  - Triple-negative breast cancer (TNBC)

- Lifestyle factors differentially affect these subtypes:
  - Obesity and estrogen exposure: Higher body fat leads to increased estrogen production, raising the risk of HR + tumors.
  - Smoking and epigenetic changes: Tobacco exposure induces DNA methylation and mutations, particularly in aggressive subtypes like TNBC.
  - Diet and gut microbiome alterations: High-fat diets can modulate gut microbiota, leading to increased systemic inflammation and altered estrogen metabolism, impacting breast cancer progression^[51]^.

3. Evaluating biomarker-driven clinical outcomes in breast cancer:

- Integrating lifestyle data with molecular markers allows researchers to predict treatment responses and survival outcomes.
- Genetic and epigenetic markers linked to chemotherapy resistance (e.g., BRCA mutations in TNBC) may be influenced by dietary antioxidants or inflammatory status.
- Metabolic profiling can reveal how insulin resistance or obesity-related markers affect endocrine therapy efficacy in HR + breast cancer.

### Case studies in MPE research on breast cancer

1. Obesity and epigenetic alterations in breast cancer: Studies have shown that obese patients exhibit hypermethylation of tumor suppressor genes (e.g., BRCA1, PTEN), potentially accelerating tumor progression. Obesity is also associated with higher insulin-like growth factor (IGF-1) levels, which activate oncogenic pathways in HR + breast cancer.
2. Alcohol consumption and breast cancer subtype risk: Chronic alcohol intake has been linked to increased DNA methylation at oncogenic loci, as well as elevated estrogen levels, raising the risk of HR + breast cancer. MPE analysis has identified alcohol-induced changes in microRNA expression (e.g., miR-21 upregulation), which promotes breast cancer invasion and metastasis^[52]^.
3. Air pollution and DNA methylation in triple-negative breast cancer: Exposure to polycyclic aromatic hydrocarbons (PAHs) and fine particulate matter (PM2.5) from air pollution induces epigenetic silencing of tumor suppressor genes, particularly in aggressive TNBC cases. Studies suggest that women living in high-pollution areas may experience earlier onset and higher mortality rates due to pollution-related oxidative stress and inflammatory signaling.

### Clinical applications of iron chelators in breast cancer therapy

Iron chelation therapy has emerged as a promising therapeutic strategy for breast cancer treatment, particularly due to its ability to target the altered iron metabolism commonly observed in cancer cells. As breast cancer cells often exhibit dysregulated iron homeostasis to support their rapid growth and proliferation, the use of iron chelators offers a novel approach to disrupt these processes. This disruption can lead to enhanced tumor cell death, improved sensitivity to traditional therapies such as chemotherapy and radiation, and potential reductions in metastasis. Several iron chelators have been studied in clinical and preclinical settings for their potential to combat breast cancer, offering insights into their mechanisms of action, efficacy, and safety profiles^[53,54]^. One of the most widely studied iron chelators is DFO, a drug originally developed to treat iron overload disorders. In breast cancer therapy, deferoxamine has shown promise by selectively targeting the excess iron in tumor cells and inducing oxidative stress, which is lethal to rapidly proliferating cancer cells. Clinical studies have demonstrated that DFO can enhance the efficacy of chemotherapy by increasing ROS production and impairing DNA repair mechanisms in breast cancer cells. Moreover, deferoxamine’s ability to sensitize tumors to radiation therapy has been highlighted in various studies, where its administration has been shown to improve tumor response to radiotherapy by increasing the DNA damage caused by radiation. Despite its potential, DFO has limitations, including its relatively short half-life and the need for parenteral administration, which may reduce its clinical applicability. However, its use in combination with other therapies is being explored to maximize its effectiveness^[55,56]^.

Another iron chelator, deferasirox, has been studied for its potential in breast cancer therapy. Unlike deferoxamine, deferasirox is an oral iron chelator, offering a more convenient option for long-term use. Research has shown that deferasirox not only reduces intracellular iron levels but also modulates various signaling pathways involved in tumor cell growth and metastasis. In preclinical models, deferasirox has been shown to inhibit breast cancer cell proliferation and enhance the anti-tumor effects of chemotherapy agents such as doxorubicin and paclitaxel. Additionally, deferasirox has been found to influence the tumor microenvironment, reducing angiogenesis and inhibiting the migration and invasion of breast cancer cells. Clinical trials investigating the combination of deferasirox with chemotherapeutic agents are ongoing, with early results suggesting that it may hold promise as a therapeutic adjunct in breast cancer treatment^[57]^. Dypyridyl and other small-molecule iron chelators have also been investigated for their ability to inhibit breast cancer cell growth and metastasis. These compounds work by binding to iron and preventing its involvement in cellular processes that support tumor progression, such as DNA synthesis and mitochondrial function. Studies have shown that dypyridyl can effectively inhibit tumor growth in various breast cancer models, with the added benefit of being able to cross the blood-brain barrier, making it a potential candidate for treating breast cancer metastasis to the brain. While these agents are still under investigation, their ability to target specific tumor types and modulate cancer cell metabolism presents a unique opportunity for the development of novel therapeutic strategies.^[58-60]^

In addition to iron chelators, other approaches that involve targeting iron metabolism are also being explored in clinical trials. One such strategy is the development of transferrin receptor-targeted therapies. As TfRs are upregulated on many breast cancer cells, they provide a potential target for targeted drug delivery systems. Iron chelators conjugated to transferrin or transferrin receptor-binding agents have been designed to selectively deliver iron-depleting therapies directly to the tumor site, sparing normal cells. This targeted approach enhances the therapeutic efficacy while reducing off-target side effects, a major challenge with conventional chemotherapy. Early-phase clinical studies of these conjugates have shown promising results, and ongoing research aims to optimize these strategies for clinical application in breast cancer.^[61-63]^ Moreover, combination therapies that integrate iron chelation with existing breast cancer treatments are gaining significant attention. For example, combining iron chelators with chemotherapy agents such as doxorubicin has been shown to potentiate the chemotherapy’s cytotoxic effects. The rationale behind such combination therapies is that iron chelators can increase the tumor’s sensitivity to chemotherapy by impairing DNA repair and enhancing oxidative stress, making it more susceptible to the DNA-damaging effects of chemotherapeutic agents. Similarly, combination with radiation therapy has demonstrated the potential to improve therapeutic outcomes by amplifying radiation-induced DNA damage through increased ROS production^[27]^.

**Figure 2.**
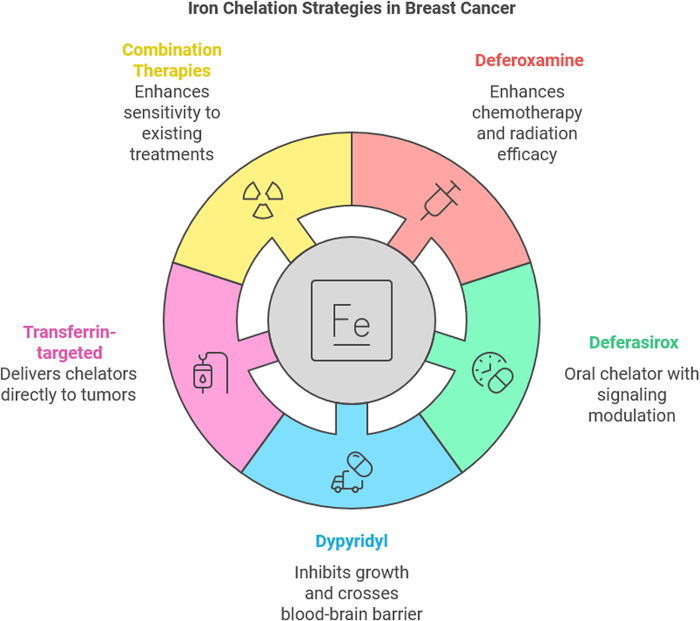
Iron chelation strategies in breast cancer.

However, the clinical application of iron chelators in breast cancer is not without challenges. While iron chelation can be beneficial for cancer therapy, there is the risk of depleting iron to a level that may impair normal cell function. Iron is a crucial element for various cellular processes, including erythropoiesis and immune cell function. Therefore, the use of iron chelators must be carefully managed to balance therapeutic benefits with potential risks such as anemia or immune suppression. Furthermore, long-term use of iron chelators may lead to the development of iron deficiency, which requires ongoing monitoring and management of iron levels in patients^[34]^. Another critical consideration is the tumor heterogeneity observed in breast cancer. Different subtypes of breast cancer, such as hormone receptor-positive, HER2-positive, and triple-negative breast cancer, may respond differently to iron chelation therapy. Tailoring iron chelation therapy to specific breast cancer subtypes is essential to optimize treatment outcomes and minimize side effects. Personalized approaches based on tumor profiling and iron metabolism markers are being explored to identify which patients are most likely to benefit from iron chelation therapy^[27]^. Figure 2 shows iron chelation strategies in breast cancer (provided by the authors).

### Conclusion

Iron chelation therapy has emerged as a promising adjunct to traditional breast cancer treatments, offering a novel approach to targeting the altered iron metabolism that is commonly observed in cancer cells. By reducing the excess intracellular iron in tumor cells, iron chelators can disrupt essential processes such as DNA replication, cellular respiration, and oxidative stress management, ultimately leading to increased tumor cell death. Several iron chelators, including deferoxamine, deferasirox, and dypyridyl, have shown encouraging results in preclinical and clinical studies, with the potential to enhance the efficacy of chemotherapy, radiation therapy, and targeted therapies. Despite the therapeutic potential, the clinical use of iron chelators in breast cancer therapy presents several challenges, including the risk of iron deficiency and the need for personalized treatment strategies based on tumor subtype and patient-specific factors. Furthermore, while iron chelation may improve treatment outcomes in certain contexts, careful monitoring and optimization of iron levels are crucial to avoid complications such as anemia and immune suppression.

## Data Availability

None.
